# Calcium/Calpain mediated regulation of cyclic nucleotide phosphodiesterases, PDE3A and PDE5, in human platelets

**DOI:** 10.1186/1471-2210-11-S1-P57

**Published:** 2011-08-01

**Authors:** Irina G Rybalkina, Masami Shimizu-Albergine, Sergei D Rybalkin

**Affiliations:** 1 Department of Pharmacology, Box 357280, University of Washington, 1959 NE Pacific St., Seattle, WA 98195-7280, USA

## Background

Calpain, intracellular non-lysosomal calcium-dependent proteinase, is activated by a number of signaling pathways that lead to the elevation of intraplatelet calcium concentrations. Physiological roles of calpain, highly expressed in platelets, include regulation of platelet aggregation and adhesion, while excessive activation of calpain in such pathological disorders as diabetes could cause changes in platelet functions, resulting in platelet hyperactivation. Platelets from diabetic patients are also characterized by resistance to nitric oxide inhibition. Here we examined if calpain could regulate major cAMP and cGMP hydrolyzing phosphodiesterases (PDEs), PDE3A and PDE5, in human platelets.

## Results

Elevation of cytosolic calcium in human platelets with A23187 as well as treatments with thapsigargin, resulted in increases of catalytic activities of PDE3A and PDE5. These changes were accomplished by their partial proteolysis. Our data showed that calpain was responsible for cleavage of PDE3A and PDE5. Calpain, activated by an increase of cytoplasmic Ca, induced limited proteolysis of cyclic nucleotide PDEs, whereas calpeptin, a calpain inhibitor, was able to block the proteolytic effects of calpain. PDE3A was very susceptible to the duration of calpain proteolysis, and prolonged exposure of PDE3A to the activated calpain led to the generation of several truncated forms of PDE3A with lower catalytic activities.

Contrary to PDE3A, calpain cleaved only a part of the N-terminal domain of PDE5, a region before the PKG- (serine-102) phosphorylation site. This truncated form of PDE5 was stable and the only one produced by calpain during its activation. Calpain-activated PDE5 also showed higher sensitivity towards inhibition by a PDE5 specific inhibitor, sildenafil.

Confocal immunofluorescence analyses of PDE5 localization in spreading platelets identified PDE5 mostly in the center, around inner actin rings. Calcium treatments changed platelets morphology and PDE5 localization, showing diffuse cellular staining.

## Conclusion

Thus our data suggest that Calcium/Calpain induced cleavage of PDE3A and PDE5 may represent a novel mechanism of regulation of cyclic nucleotide PDEs in platelets during development of diabetes and cardiovascular dysfunctions.

